# Beamless Metal Additive Manufacturing

**DOI:** 10.3390/ma13040922

**Published:** 2020-02-19

**Authors:** Mohammad Vaezi, Philipp Drescher, Hermann Seitz

**Affiliations:** 1 Mechanical Engineering Department, Babol Noshirvani University of Technology, Babol 47148-71167, Iran; 2 Department of Mechanical Engineering and Marine Technology, Chair of Microfluidics, University of Rostock, 18059 Rostock, Germany; philipp.drescher@uni-rostock.de (P.D.); hermann.seitz@uni-rostock.de (H.S.); 3 Department Life, Light & Matter, University of Rostock, 18059 Rostock, Germany

**Keywords:** metal additive manufacturing (AM), beamless metal AM, non-beam metal AM

## Abstract

The propensity to manufacture functional and geometrically sophisticated parts from a wide range of metals provides the metal additive manufacturing (AM) processes superior advantages over traditional methods. The field of metal AM is currently dominated by beam-based technologies such as selective laser sintering (SLM) or electron beam melting (EBM) which have some limitations such as high production cost, residual stress and anisotropic mechanical properties induced by melting of metal powders followed by rapid solidification. So, there exist a significant gap between industrial production requirements and the qualities offered by well-established beam-based AM technologies. Therefore, beamless metal AM techniques (known as non-beam metal AM) have gained increasing attention in recent years as they have been found to be able to fill the gap and bring new possibilities. There exist a number of beamless processes with distinctively various characteristics that are either under development or already available on the market. Since this is a very promising field and there is currently no high-quality review on this topic yet, this paper aims to review the key beamless processes and their latest developments.

## 1. Introduction

There has been a trend in metal additive manufacturing (AM) in recent years and much of the reports on AM are about the growth in metal-AM and its significant impact on design and rapid manufacture of geometrically complicated high-end parts. A recent Wohlers Report [1] demonstrates a significant growth in the metal-AM market so that an estimated 1768 metal-AM systems were sold in 2017, compared to 983 systems in 2016, an increase of approximately 80%. The main benefits of metal-AM in comparison with conventional manufacturing are a shorter value chain, reduced production cost and lead times for intricate parts, greater design freedom and customization, and minimal waste of materials.

**Figure 1:**
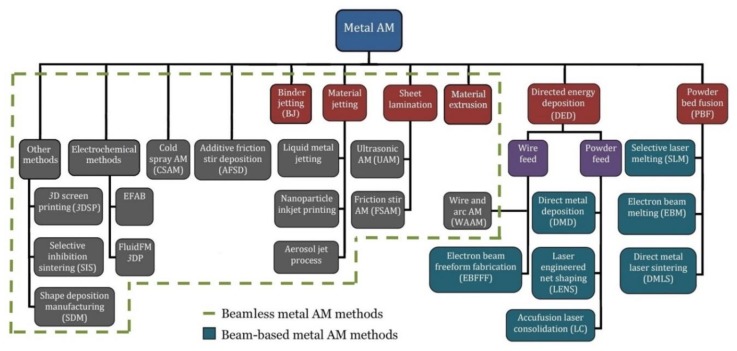
Classification of the main current metal AM processes, including beam-based and beamless techniques.

The current highlights of metal AM are divided into two main categories: beam-based and beamless techniques (Figure 1). As classified by American Society for Testing and Materials (ASTM), powder bed fusion (PBF) and directed energy deposition (DED) methods represent the key beam-based approaches for fabricating metals and alloys [2]. Comprehensive reviews of the beam-based metal-AM systems have been presented in [3,4,5,6]. EBM and SLM, the main representative of the PBF method, are currently dominant metal-AM processes because of the superior mechanical properties of the printed highly demanding functional parts. In these beam-based techniques, the powder/beam interactions result in sophisticated physical phenomena including: melting, dynamic melt flow, and rapid solidification that results in highly orientated, columnar grains with anisotropic mechanical properties [3]. The high thermal stress and rapid solidification can also lead to detrimental residual stress and defects such as delamination and severe part distortion. In addition, the beam-based metal-AM systems are energy inefficient, and suffer from the high costs of technology and operations as well as constraints on speed, precision and surface quality. Moreover, it is quite challenging to process non-weldable metals through beam-based metal-AM systems [7,8].

Beamless metal AM techniques (known as non-beam metal AM) are being employed effectively to overcome the above limitations of the beam-based systems. There exist a number of beamless processes with various distinct characteristics that are either under development or already available in the market. These approaches provide different opportunities in terms of process economics, mechanical properties, achievable geometries and surface quality.

**Figure 2:**
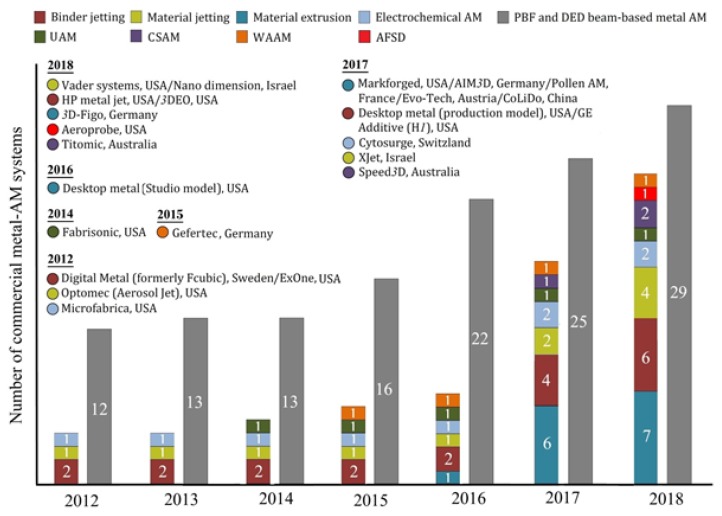
Growth in beamless metal-AM systems comparing with beam-based systems (column numbers indicate the number of most common commercially launched systems), data acquired from the Scopus database, and the Wohlers Report [1].

Beamless metal AM has been able to draw the attention of both the research community and industry. The growth in beamless research over the past five years is reflected in the number of publications [9]. In addition to the research community, there has been a significant growth in the beamless metal AM market as presented in Figure 2.

As seen in Table 1, the beamless systems are additive processes in which material bonding within and between layers occurs without using laser/electron beam melting. Liquid metal jetting (a type of material jetting technology), wire and arc AM (WAAM), and shape deposition manufacturing (SDM) approaches are based on material melting, similar to beam-based techniques. However, they use energy sources other than laser or electron beam for melting of materials. Some of the beamless methods including binder jetting (known as 3D printing), material extrusion, nanoparticles inkjet printing, selective inhibition sintering (SIS), aerosol jet, and 3D screen printing use bulk sintering in the furnace for layer bonding. The extrusion-based systems can massively reduce production costs, while binder jetting can achieve higher print resolution and surface finish than the beam-based systems. More important, the printed metal parts using this class of metal AM systems possess remarkably lower residual thermal stress than beam-based systems since the green parts are sintered after printing.

EFAB and FluidFM 3D printing technologies use electrochemical deposition methods that outperform the beam-based systems in terms of resolution and accuracy. Conversely, ultrasonic AM (UAM), additive friction stir deposition (AFSD), friction stir AM (FSAM), and cold spray AM (CSAM) processes use solid state thermo-mechanical bonding without the need for a protective print environment for printing mega scale parts which are not viable through beam-based systems [9]. UAM enables fast and scalable manufacturing for a wider range of engineering alloys, multiple materials and composites, and parts with embedded electronics due to its low operation temperature. CSAM has a very high deposition rate (almost 10 times faster than SLM) with no limitation in build size that makes this process well-suited for industrial scale production of near net shape large parts.

A comprehensive review of the key beamless metal AM systems and the current state of their research and technology are presented in this paper. The review outlines the principle of each beamless metal-AM processes followed by disseminating the efforts and recent progresses. Techniques that either have not been widely adopted (e.g., SDM and SIS) or their application field is mainly confined to writing (such as aerosol jet that needs laser beam for true 3D fabrication) are not addressed in this paper.

This review is based on Scopus and Google Scholar searches. Articles published over the period 2012–2019 have been selected and the full papers and other reviews have been analysed. Furthermore, Wohlers Report 2018 [1] and various internet sources have been used to analyse the market situation and future industrial trends in metal AM.

## 2. Key Processes

### 2.1. Material Jetting Processes

Material jetting which includes processes like liquid metal printing, nanoparticles inkjet printing or aerosol jet process (see Figure 1) is an AM process in which liquid droplets of build material are selectively deposited and become solid via cooling (e.g., by crystallization or vitrification), chemical changes (e.g., cross-linking of a polymer), or solvent evaporation [2,10,11]. Post-processing procedures such as sintering might be included to achieve near fully dense parts.

The material jetting process was initially used for printing parts out of wax [12] and polymers [13]. The material jetting process has also been widely used to print a variety of functional bio-constructs and tissues by patterning living cell-loaded materials and direct cell printing [14]. Orme et al. [15] and Yamaguchi et al. [16] first demonstrated 2.5D jetting of molten AA2024 aluminum (density 98.5%) and true 3D metal jetting of a molten fusible alloy (Bi-Pb-Sn-Cd-In alloy), respectively.

A number of research works have been conducted on droplet-based printing of low/medium stiffness metals such as Sn63-Pb37 alloys [17,18,19,20], aluminum [21,22,23], copper [24], tin, various solders [25] using nozzles typically between 50 to 200 μm in diameter.

Development of new jetting systems with higher print quality has been the main focus of recent research. In inkjet printing, the drop diameter normally ranges from one to two times that of the nozzle [26]. Droplets as small as around 60% [27] and 55% [28] of the nozzle diameter could be printed using a new pneumatic DOD and impact-driven ejection, respectively. To improve uniformity and accuracy of metal droplets, a pneumatically actuated inkjet printing system composed of a star-shaped nozzle was proposed for the generation of liquid metal microdroplets in the nano- to picoliter range [29,30]. High resolution pillars and microstructures with wall thickness of 25 µm could be printed using laser-induced forward transfer (LIFT) print heads that use laser beam to melt a thin metal layer for creating femtoliter droplets [31]. Electromagnetic print heads were also developed to eject uniform metal droplets at lower cost and higher speed [32]. The electromagnetism driven printing uses a piston that is accelerated by the magnet force to create droplets. Electrohydrodynamic printing technology (known as e-jet printing) was used by Han et al. [33] for high resolution printing of a double layer aluminum scaffold with uniform struts as fine as 100 µm. In e-jet, a high voltage is applied between a pneumatic-actuated nozzle and the ground electrode placed under the metal substrate to print molten metals at remarkably higher resolution than normal pneumatic jetting system.

The limited crucible volume is the main obstacle for further commercialization of liquid metal 3D printing. To overcome this problem, a wire-based feeding system was proposed by [34] for generation of continuous droplet stream of a low-melting-point alloy. The first liquid metal 3D printer was commercialized by Vader Systems under the trade name of “MagnetoJet” that uses a method very similar to that proposed several years ago by International Business Machines Corp (IBM, patent no. US5377961A). It uses magentohydrodynamic (MHD) actuation [35] integrated with a wire-based feeding system. Recently, Spiller and Fleischer [36] used a commercial ARBURG freeformer machine (originally developed for melt 3D printing using standard plastic granules) in the metal injection molding (MIM) process chain instead and proposed a new approach for droplet-based AM of sintered metal components. Their proposed ARBURG metal freeforming could reach properties very close to MIM; tensile strength: 95.7–99.2% of MIM produced metal parts.

Molten metal jetting is limited to low/medium melting temperature metals as for other metals a high-temperature resistant printing system is required to keep the metal molten during the jetting process [37]. XJet (Rehovot, Israel) has recently commercialized a low-temperature metal 3D printer that uses its proprietary NanoParticle Jetting™ (NPJ) technology. It prints nanoparticle inks of both build and support materials in ultrafine layers simultaneously. High temperature of the build chamber results in evaporation of the liquid content, leaving dense layers of materials with chemical composition similar to conventionally produced parts. The 3D printed green parts are then subjected to sintering process to achieve final dense metal parts, and support materials are easily removed.

### 2.2. Binder Jetting

Binder jetting (BJ), known as three-dimensional printing (3DP), was first developed at the Massachusetts Institute of Technology [38]. Binder jetting has demonstrated the capability of printing complex parts from a variety of materials, including ceramics [39], metals [40], shape-memory alloys (SMA) [41,42], polymers [43], polymer/ceramic [44] and drugs [45]. In the case of metallic powder, the printed green body is placed in a furnace to remove the binder and sinter the powder particles for densification. However, the final sintered parts possess relatively low density since powders are loosely deposited in the binder jet process. Therefore, other techniques such as infiltration (using a metal with lower melting point) and/or hot isostatic pressing (HIP) [46,47] are normally applied after sintering to enhance the density of the final part.

Binder jetting has been successfully used for printing of different metals such as stainless steel [48], titanium [49,50,51,52,53], biodegradable iron–manganese alloys [54], WC-CO hardmetals [55], superalloys [56,57,58], cobalt-chrome [59], magnetic materials [60,61,62], and high purity copper [63] that is challenging to process using beam-based AM technologies due to its optical reflectivity and high thermal conductivity.

**Figure 3:**
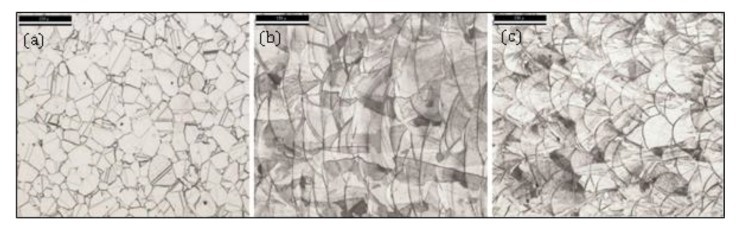
Optical microscopy images of 316L samples in etched condition (Glyceregia etchant): (**a**) BJ 50× (**b**) EBM 50×; (**c**) DMLS 50×. Scale bars are 150 µm [70].

Microstructural analysis and investigation of mechanical properties of the binder jetted metals has been a focal research topic [64,65]. Basically, the metal BJ process generates a relatively fine equiaxed grain microstructure, while the EBM and DMLS processes are conducive to comparable columnar grain microstructures as illustrated in Figure 3. Fatigue performance of the printed nickel-based superalloy 625 part could be improved by mechanical grinding of its surface, surpassing that of the cast alloy [66]. Steel Parts with tailored material gradients have been 3D printed using nanoparticle carbon black ink [67]. Sheydaeian and Toyserkani [68] developed a novel system for printing porous titanium parts by selective encapsulating of the sacrificial paraffin particles inside the powder layers during the binder jetting process. Porous biodegradable scaffolds have been binder jetted from Fe-Mn-Ca/Mg alloys with controlled in vitro degradation and cell attachment [69].

The main limitation of metal binder jetting is the relative density of metal parts (95–97%), which can be compared with a typical density of > 99% with laser-based powder bed systems. A number of research works have been conducted to investigate the effect of process parameters (such as layer thickness, binder saturation, drying after printing each layer) and part orientation [58,71], powder particle size and distribution [53,72,73,74], and post-processing [47] on density, and mechanical properties. The influence of layer thickness, powder particle size and sintering profiles in the binder jetting of IN718 superalloy was studies by Turker et al. [58]. Shrestha and Manogharan [75] used the Taguchi method to determine the optimum process parameters for improving transverse rupture strength of sintered SS 316L samples. Binder saturation level and feed-to-powder ratio were demonstrated as the most critical parameters, affecting binder/powder interaction and mechanical properties of the sintered parts. The use of bimodal powders has been demonstrated to be an effective approach to improve the packing density of powders, and thus surface finish [76] and final sintered density [77]. Kumar et al. [47] demonstrated that the use of HIP can improve the final copper part density from 92% (as-sintered) to 99.7% of theoretical density. HIP of powder bed fusion AM processes typically results in a strength drop due to a significant increase in the grain size. However, HIP improves the strength of BJ parts, which indicates that compared to grain coarsening, the improvement in porosity is the dominant factor in determining strength [47].

The selection of a binder material system determines green part strength and affects the sintered density and shrinkage [78]. Solvent-based polymeric binders are commonly used binders in commercial metal binder jetting systems. The use of the polymeric binders can add complexity and limitations to the sintering process and adversely affect the final mechanical properties. Functional inks containing metal nanoparticles have been proposed to replace polymeric binders with the aim of enhancing densification. Silver [79] and copper [80] parts printed using the nanoparticle ink were significantly enhanced in sintering quality than those printed using the polymeric binder system, in particular parts with thin or fine features with high aspect ratios. Particle-free metal salts with and without polymer binder were proposed [81] as an alternative approach due to difficulties in preparation and ink jetting of metal nanoparticle inks such as printhead clogging, particle sedimentation, and surface oxidation of nanoparticles during storage. Various existing inorganic metal salt solutions (e.g., copper nitrate hydroxide and copper sulfate) can be jetted, and convert to metal particles upon deposition via decomposition [81].

### 2.3. Material Extrusion

**Figure 4:**
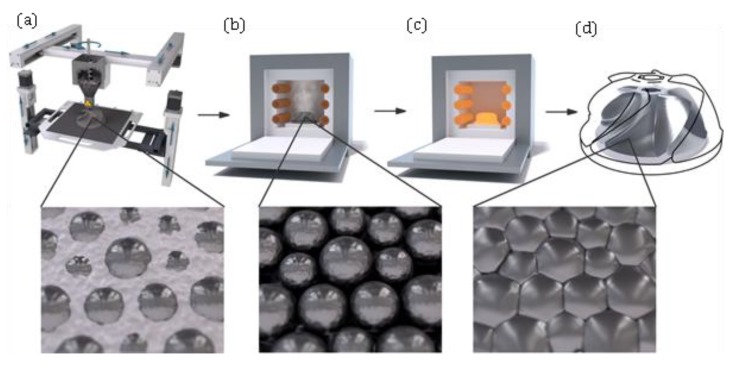
Process scheme of an extrusion-based system: (**a**) 3D printing of the green part (**b**) debinding of the green part in a debinding furnace; (**c**) sintering of the resulting brown part in a high-temperature furnace; (**d**) final metal part [85].

Material extrusion refers to the AM techniques that use an extrusion method for dispensing build material selectively. The process known under the trademarked name Fused Deposition Modelling (FDM) technology, also known as Fused Layer Modeling (FLM) or Fused Filament Fabrication (FFF) [82]. Figure 4 depicts extrusion-based metal 3D printing process schematically. As can be seen, the printed green part needs a consecutive debinding and sintering process (like BJ method) in order to remove the binding agent and fuse the particles together for the final part.

The main advantage of FDM is its low technology and operation cost as it does not require expensive components such as lasers or electron sources and vacuum pumps in beam-based machines. Another advantage is the ability to build components combining both thermoplastics and metals within the same build which is not possible with other direct metal systems [83]. The FDM technology can also achieve build rates that are much higher compared to sintering and melting technologies [84].

Basically, there are three different approaches for extrusion-based AM. Material extrusion with plungers, screw-based, or filament-based. Metal extrusion AM is mainly used by screw-based or plunger-based approaches since metal filaments present multiple problems mostly due to their mechanical properties such as brittleness.

Various material extrusion approaches have been developed with the aim of direct metal 3D printing. Greulich et al. [86] developed a process called multiphase jet solidification (MJS). The 3D fiber deposition (3DF) is a plunger-based technique that allows the development of metallic parts with accurately controlled pore size, porosity and interconnecting pores [87]. The material is a composite that consists of a metal powder and a binder or rather dispersant. Mireles et al. [83] modified an FDM 3000 system to deposit low melting eutectic Bi58Sn42 and non-eutectic Sn60Bi40 materials for electronic circuitry applications amongst other things.

Powder-binder mixtures are currently the most promising solutions for extrusion-based AM [88]. Metal or ceramic powders are combined with a polymer and form a composite, either in pellet form or as a filament that can easily be extruded [89]. The granulate has the advantage of containing a higher metal content than filaments (approx. 55 vol.% and 30 vol.%, respectively). Recent studies have shown that filaments based on stainless steel 316L [90] and copper [91] can be processed successfully. However, Gong et al. [90] found that the 316L parts feature lower yield strength, UTS, and elastic modulus compared to AISI type SS 316L as well as laser melted SS 316L part. This is induced by the equiaxed grains and austenitic microstructure of the parts. A filament based on copper and a binder system based on paraffin wax, low density polyethylene, and stearic acid (PW–LDPE–SA) was developed and tested by Ren et al. [91].

**Figure 5:**
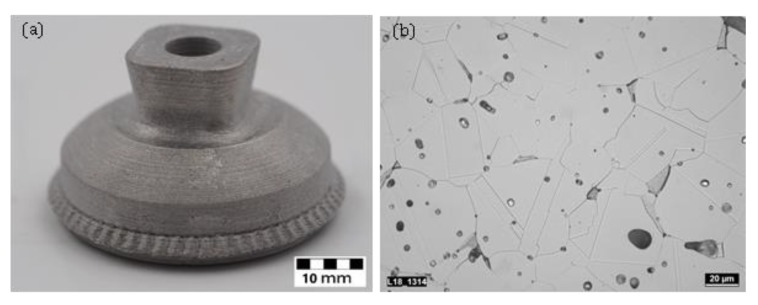
(**a**) CEM printed and sintered 316L part and using conventional MIM feedstock Catamold (BASF, Ludwigshafen, Germany) (**b**) optical microscopy of CEM printed 316L part showing microstructure and inclusions of carbon and foreign particles comparable to conventional manufactured MIM parts.

A screw-based approach using conventional metal injection molding (MIM)-feedstock was introduced as composite extrusion modelling (CEM) by Lieberwirth et al. [85]. The final parts featured a compression strength of 2345 MPa and a density of 7.47 g/cm^3^ which is very close to the typical value for the density of 7.67 g/cm^3^ specified conventionally processed MIM parts. A CEM printed test part made from 316 as well as an optical microscopy of the microstructure are depicted in Figure 5.

Another plunger-based material extrusion approach is termed 3D gel-printing (3DGP), which selectively deposits a slurry layer-by-layer [92]. The 3DGP process is based on the in-situ polymerization of organic monomer binder and combines it with FDM. Recent approaches aim at printing binder-free metals. Kim et al. [93] developed a volumetric metal 3D printing system that can dissolve and eject binder-free molten Sn40Pb alloy materials. Another approach, which can be classified as filament-based extrusion, is by utilizing bulk metallic glasses (BMG) that show high mechanical properties. BMGs exhibit a supercooled liquid behavior, which can be used in FDM under similar conditions to those in thermoplastics. The supercooled liquid can be extruded in a similar fashion as thermoplastics [94]. In this case, there is no need for post-processing like a sintering process to remove a binding agent.

### 2.4. Cold Spray AM (CSAM)

Cold spray (CS) process was originally developed as a solid-state coating method for improving surface properties [95]. In CS process (known as dynamic gas cold spray) a supersonic gas jet (normally velocity of 300 to 1200 m/s) is used to accelerate powdered materials (diameter of 1–50 μm) through a de-Laval or similar converging/diverging nozzle and spray them onto a substrate. Deposition is achieved through local metallurgical bonding and mechanical interlocking which are caused by localized plastic deformation at the inter-particle and particle-substrate interfaces [96,97]. As there is no melting and solidification in CS, the process does not suffer from common deleterious defects in high-temperature deposition processes such as high porosity, oxidation, residual thermal stress, meting-induced phase transformation and re-crystallization [96].

**Figure 6:**
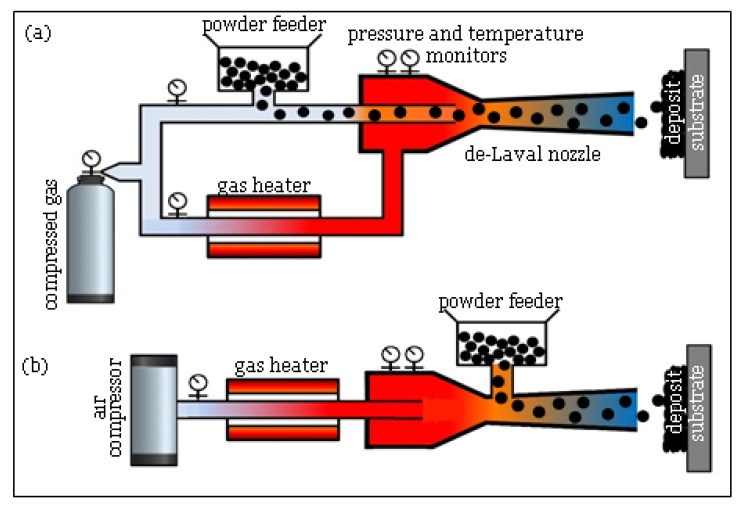
Schematic view of (**a**) high and (**b**) low pressure CS.

As illustrated in Figure 6, there are two types of CS design according to the pressure of the propulsive gas: high pressure CS in which the working gas is nitrogen or helium at pressures above 1 MPa, and low-pressure CS that uses compressed air at pressure below 1 MPa. A wide range of pure metals can be deposited using high pressure CS, while low pressure CS is used for spraying a mixture of limited metal/ceramic powders [98,99,100]. Various aspects of both processes and their applications have been discussed through some comprehensive review papers [9,96,99,101]. The term ‘cold spray’ normally refers to high pressure CS. Hereafter, this nomenclature is followed through the paper as well.

CSAM of Al and Cu alloys have been widely investigated due to their excellent deformability, and thus cold-sprayability. Dense bulks from Cu and Cu alloys (Cu-Ag, Cu-Ag-Zr) with good interface bonding and mechanical properties have been printed using optimized CS with helium gas [9]. Post annealing could increase tensile strength of the printed bulks by 34.2% (from 125 to 168 MPa) and the interfaces between particles disappearing after annealing [9]. However, annealing can effectively reduce hardness [102]. As-sprayed Cu has poor ductility with elongation below 0.4%, while post annealed samples at 400 °C had elongation similar to pure Cu bulk [103]. Al deposits have slightly lower compactness than Cu deposits due to its lower density, although Al particle can be easily deposited [104,105,106]. The influence of post-annealing and solutionizing on mechanical properties of Al6061 and Al7075 deposits were studied by Rokni et al. [105,106,107].

The relatively high strength of stainless steel makes it difficult to print dense parts. Coddet et al. [108] studied the microstructure and mechanical properties of 304L stainless steel deposits. As-printed parts were fairly dense (porosity of 1.3%) with tensile strength of 525 MPa and a brittle fracture nature with elongation of about 7%. The annealed parts at 400 °C had increased tensile strength of 629 MPa without improved ductility.

It is quite challenging to cold spray dense Ti alloys parts. The CSAMed Ti alloys exhibit a relatively high level of porosity due to their active characteristics and reactions with surrounding air [109]. Wong et al. [110,111] investigated the influence of particle morphology and size distribution on CSed pure Ti and Ti-6Al-4V. Dense Ti deposits CSed (using nitrogen) with high tensile strength of 800 MPa and very poor ductility of (approx. 0.01%) was reported by Jahedi et al. [112]. According to the study by Vo et al. [113], the porosities of the CSed Ti-6Al-4V deposits using helium gas was lower by one order of magnitude than that nitrogen gas due to higher level of particle acceleration. The use of in-situ peening technique [114] (i.e., mixing of build powder with large ceramic powders), could result in dense Ti and Ti-6Al-4V deposits using cheaper nitrogen gas due to the “tamping effect” of CS [115].

It is very difficult to deposit dense parts from superalloys even using CS at high gas temperatures [116], so there has been a great focus on investigation of the post-heat treatments routes. It has been shown that high temperature heat treatment as well as the use of helium gas can effectively enhance properties of the CSAMed superalloys [117]. Levasseur et al. [118] proposed a pressureless sintering process to decrease porosity of Inconel 718 deposits from 2.5% to 0.2% and improve flexural strength and ductility.

### 2.5. Additive Friction Stir Deposition (AFSD)

**Figure 7:**
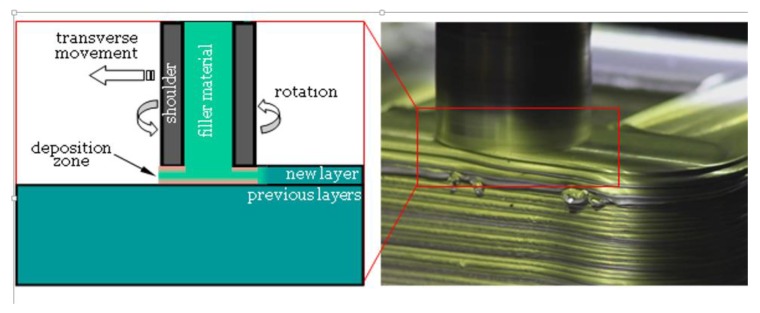
Schematic view of AFSD process (**left**), AFSD head in operation (**right image**, courtesy of Aeroprobe Corporation, USA).

This process is a solid-state metal AM process that uses heat and friction to bond materials layer-by-layer (Figure 7), developed by MELD manufacturing.

The AFSD involves a hollow rotating shoulder, through which the feed material is delivered in the form of either a solid rod or powder [119]. The rapid rotation of the hollow shoulder generates heat through dynamic contact friction at the shoulder/material and material/substrate interfaces [120]. The heated and softened material bonds with the substrate through plastic deformation at the interface. Post-heat treatment of the as-printed parts is not necessary due to low porosity and residual stress; however, the parts normally need post-machining/surface finishing.

The peak temperature of AFSD process normally ranges from 60 to 90% of the melting temperature [121]. However, a low shoulder transverse speed or a very high shoulder rotation frequency may result in adiabatic heating during deposition, and thus local melting. So, appropriate selection of process parameters is critical to prevent such phenomena and minimize defects [9]. AFSD provides important advantages of friction stir welding and friction stir processing such as the capability to process non-weldable alloys, and wrought microstructure formation [122,123]. In particular, the printed parts using AFSD have shown isotropic mechanical properties due to dynamic recrystallization and refined grains.

Multiple materials such as Nb/Cu and Ta/Cu have been successfully printed using AFSD process. No interfacial delamination was observed when applying a high shear stress at the interface through bending [119]. Also, customized reinforced Al and Mg-based alloys such as Al-Mo, Al-W, and Al-SiC have been fabricated with loading up to 30 Vol.% [119]. In addition to Al alloys, AFSD of high strength alloys such as nickel-based superalloys [121] and titanium alloys is possible.

### 2.6. Sheet Lamination Processes

#### 2.6.1. Ultrasonic AM (UAM)

**Figure 8:**
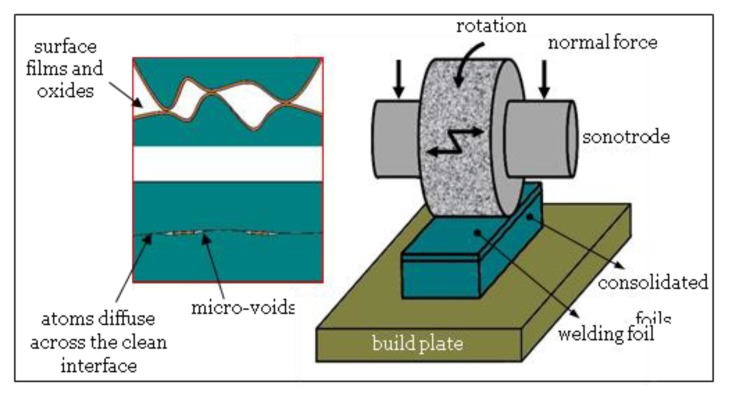
Schematic view of UAM process.

Ultrasonic additive manufacturing (UAM), known as ultrasonic consolidation (UC), is a hybrid additive/subtractive process that involves the layer-by-layer ultrasonic scrubbing of similar or dissimilar metal foils in the solid state with periodic cutting operation (often end-milling) to fabricate desired part geometry [124]. The process uses ultrasonic vibrations in a textured sonotrode to create a friction-like relative motion between two surfaces that are held together under pressure (Figure 8). This action, in turn, causes shearing and plastic deformation between asperities of the opposing surfaces, which disperses surface oxides and contaminants. As the asperities collapse, metal-to-metal contact is increased, creating solid-state metallurgical bonding between the parts through atomic diffusion. The UAM process can be used for fabrication of pure metals, alloys, and dissimilar material parts using high mechanical loads and high power ultrasonic [125].

The common defect in UAM-printed parts is micro-voids that are produced along the interfaces between bonded layers due to foil surface roughness, insufficient or excessive welding energy, and metal foil positioning inaccuracies [126]. Although printed metal parts using UAM possess high tensile/compressive strength and good wear resistance, the strength in build direction is markedly lower than the other directions [127]. Anisotropic mechanical behavior has been reported for printed Al6061 parts [128]. HIP has been found to be an effective post-treatment approach for eliminating the brittle fracture and improving the strength and ductility of UAM printed AL parts in the build direction [129,130].

**Figure 9:**
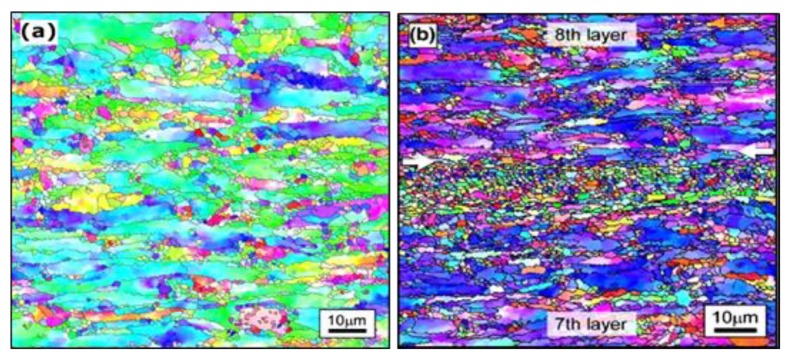
(**a**) The Inverse Pole Figure (IPF) map of as-received original AA 3003 alloy foil in which the grains are elongated along the rolling plane, and fine equiaxed grains are observed between the elongated grains, (**b**) The IPF of the VHP-UAM sample in which the interface region is represented by the white colored arrows. Many grains in the both top and bottom bulk regions are elongated along the rolling plane of the original tape, and fine grains are seen between these elongated grains, while all grains in the interface region are fine and equiaxial [132].

The use of very high power UAM has been shown to improve elastic properties of the printed AL alloys [131]. Sridharan et al. [128] used very high power (VHP) UAM (9 kW) instead of low power (1.5 kW) for fabrication of 6061 Al alloy and could achieve a complete metallurgical bonding across the interfaces without any discernable voids; however, poor mechanical properties were observed in the build direction. The microstructural analysis of VHP-UAMed AA 3003 alloy revealed that the top and bottom bulk regions consist of the original elongated grains and fine equiaxed grains (very similar to the microstructure of the as-received foils), whereas the interface regions consist of only fine equiaxial grains due to the onset of recrystallization during VHP-UAM [132] (Figure 9).

The low operation temperature and the plastic flow of metals provide the opportunity to embed and/or encapsulate various electronic devices in to the metal matrix through the UAM process. Smart material structures could be printed by embedding fibres, conductor, dielectric, and nano-fibres of NiTi shape memory alloy into a metal matrix [133]. Carbon fibres [134], fibre Bragg grating (FBG) [135], polymer-coated and uncoated optical fibres [136], metal-coated optical fibres [137], and NiTi shape memory alloy fibres [138,139] could be successfully embedded into Al matrix with sufficient bond strength in the welded interfaces of the metal foils while retaining their functionality.

Moreover, solid-state nature of UAM allows joining of dissimilar metals without the formation of brittle intermetallics as seen in powder-bed fusion processes. High performance multi-material parts such as Al/Cu, Ni/Stainless steel, and Al/Ti are routinely produced using high power 9KW UAM [132]. Hehr et al. [140] combined UAM and metal matrix composite (MMC) materials to develop a novel functionally graded materials (FGM) for aerospace applications.

#### 2.6.2. Friction Stir Additive Manufacturing (FSAM)

This process is the other type of friction-based technique that is a sheet lamination process. In FSAM, friction stir welding is used to bound stacked multiple metal sheets. A special friction pin is penetrated into the stacked sheets and then a welding line is created throughout the sheets by rotation and traverse movement of the pin. Large parts are produced by repeating the sheet stacking and friction welding, and further machining process to form the accurate external shapes. So, both AFSD and FSAM are intensive techniques as parts are printed through a severe plastic deformation at high temperatures, yet solid state. The main difference is that the FSAM is a hybrid additive/subtractive process that produces microstructures with cyclic variations [141,142], while in AFSD all layers are subjected to the same type of plastic deformation and friction-induced heating, and thus it can produce a much more homogeneous microstructure. Although both UAM and FSAM are hybrid sheet lamination processes, they differ in build layer thickness. FSAM uses thick metal sheets, for example 1.7 mm sheets of Mg-based WE43 alloy [141], while much thinner metal foils with thickness of ~0.1 mm are used in UAM process. Table 2 compares the three solid-state metal additive processes in detail.

### 2.7. Wire and Arc AM

Wire and arc additive manufacturing (WAAM) is becoming more popular due to its high deposition rate, low production cost and the capability for fabricating large-scale components [143]. Contrary to blown powder processes such as laser cladding (CLAD) or laser metal deposition (LMD), the WAAM technology is considered a wire feed process. The wire is welded using an electric arc as a heat source to build near net shape parts in layers. The raw part must often undergo CNC milling to get its final condition. WAAM is also known as shaped metal deposition (SMD) or Rapid Plasma Deposition™ (RPD™).

WAAM is a promising technology for producing large metal components (up to several meters) with moderate complexity. Specifications of this technology can be roughly defined in a high build rate of up to 2500 cm^3^/h or 5–6 kg/h [144]. Many different metals and alloys can be processed with the WAAM process. The microstructure and mechanical properties of various metals, including titanium, aluminum, nickel, steel and other intermetallic materials fabricated by the various WAAM processes have been reviewed extensively [144,145,146,147]. In general, printed metal parts exhibits anisotropy in the mechanical properties and low surface quality in comparison to other AM technologies [144].

**Figure 10:**
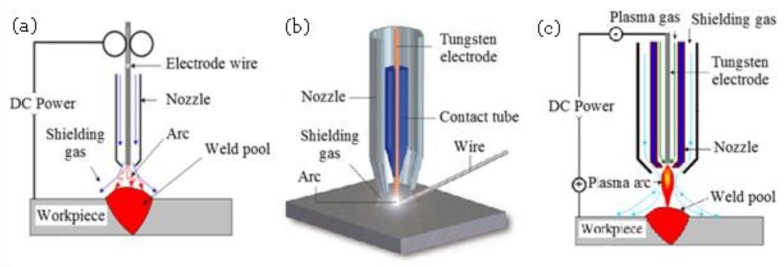
Schematic view of the (**a**) GMAW, (**b**) GTAW, and (**c**) PAW process [149].

Furthermore, WAAM can be distinguished between the use of different heat sources. The WAAM process can use gas metal arc welding (GMAW or MIG), gas tungsten arc welding (GTAW or TIG) or plasma arc welding (PAW). Figure 10 shows schematic diagrams of the mentioned processes. In general, the deposition rate of GMAW-based WAAM is 2–3 times higher than that of GTAW-based or PAW-based methods [145]. However, the GMAW-based WAAM is less stable and generates more weld fume and spatter due to the electric current acting directly on the feedstock. The energy efficiency of arc welding processes in GMAW or in GTAW can be as high as 90% in some circumstances [145]. GTAW and PAW arc welding technologies have demonstrated to be more reliable processes for WAAM with fewer problems of sputtering, excessive heating, distortion or porosity than GMAW. However, both technologies do not feed the wire coaxially which leads to process variations when changing the welding direction causing extreme sensitivity to the arc length [144,148]. There are four main approaches of metal transfer in GMAW including: globular, short-circuiting, spray and pulsed-spray, each has distinct features [145].

There are different arc welding technologies that are being used. Motion can be provided either by robotic systems or by computer numerical controlled gantries. TopTIG, for example, is a robotic GTAW welding technology with integrated wire feeder that distinguishes itself with the design of the welding torch. Cold metal transfer (CMT) is a modification of the short-circuit GMAW process which is based on controlled dip transference [149]. PAW can be considered a conventional welding technology, while CMT and TopTIG are evolutions of GMAW and GTAW, respectively.

### 2.8. Electrochemical AM Processes

**Figure 11:**
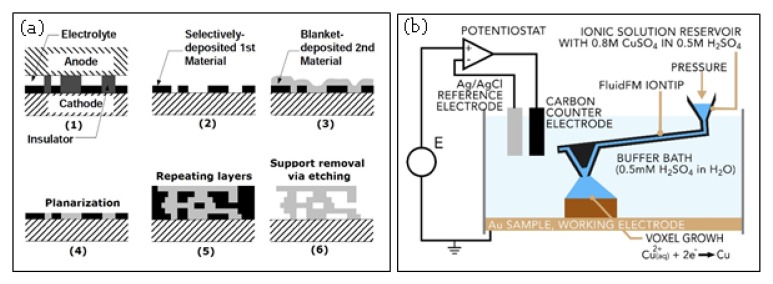
Schematic view of (**a**) EFAB [150] and (**b**) FluidFM printing cell. the iontip dispenses the electrolyte into a buffer bath. At the working electrode, Cu^2+^ ions are deposited as solid copper [151].

There currently exist two electrochemical metal-AM processes, namely, electrochemical fabrication (EFAB) and FluidFM 3DP. The EFAB (a trademark of Microfabrica, Van Nuys, CA, USA) method uses electrochemical deposition and subtractive planarization in a layer-by-layer process to build 3D micro-objects [150]. It involves three main processes in each layer: electroplating of sacrificial support material using a proprietary method called “instant masking”, electroplating of build material, and planarization, respectively. Figure 11a shows the steps for fabrication of metal parts using the EFAB method.

The EFAB has been developed for true 3D manufacturing of metal microparts with features as fine as 20 μm and accuracy of 2 μm. So far, limited metals have been developed by Microfabrica to be processed through EFAB, including: VALLOY-120^TM^, EDURA-180^TM^, copper, and biocompatible noble palladium. More information about the material properties can be found in the literatures [150].

FluidFM 3DP process is another electrochemical technique developed recently by Cytosurge AG (Opfikon, Switzerland). It combines electrodeposition, scanning probe microscopy (SPM), and precise liquid ink dispensing [151]. The system uses atomic force microscopy (AFM) cantilevers with a microfluidic channel and a hollow tip. A microfluidics control system applies a pressure to a reservoir containing the electrolyte solution to push it through the cantilever and out of the tip (Figure 11b). The tip is part of the printing head that has 3D movement inside buffer vat. The metallic ions are converted to solid metals by applying an appropriate potential to the build substrate. There is a real-time feedback to detect the tip deflection. As a voxel reaches completion, the top side of the voxel interacts with the tip, exerting force that results in a few nanometer deflection of the cantilever. When specific amount of deflection is reached, the voxel is considered as printed and the tip is moved to the next voxel.

### 2.9. 3D Screen Printing (3DSP)

This process has been developed by researchers at Fraunhofer Institute for Manufacturing Technology and Advanced Materials (IFAM), Branch Lab Dresden (Germany) [152]. It uses the classical screen-printing technique through an adapted layer-by-layer process to stack multilayers of the desired layout on top of each other and create metal parts. For each layer, a printing screen is fabricated using a photolithographic process. Metal paste containing metal particles and organic binder/additives is pushed through the screen using a squeegee being moved over the screen. Each printed layer (normal thickness of 5–15 µm) is subsequently dried in a furnace. These steps are repeated to create the final green part that is then transferred into an oven for debinding and sintering.

The metal paste formulation and its rheological behavior is the key parameter to achieve high accuracy detailed parts. The process has limitation in terms of solid particle loading (up to 50 Vol.%) as too high particle concentration can result in undesired shear thickening behavior. A wide range of metals such as stainless steel, copper, titanium, and hard metals have been successfully fabricated with surface roughness of Ra = 5–10 µm [153]. Also, the printing paste can be changed during the print process while the printing screen is kept the same, allowing for fabricating the multiple materials. A ceramic (ZrO_2_)/metal (17-4-PH) sandwich structure, and copper/ceramic 3D patterns with line width of 150 µm could be printed and sintered without cracks [153].

## 3. Impact and Applications

### 3.1. Processes Comparison

**Figure 12:**
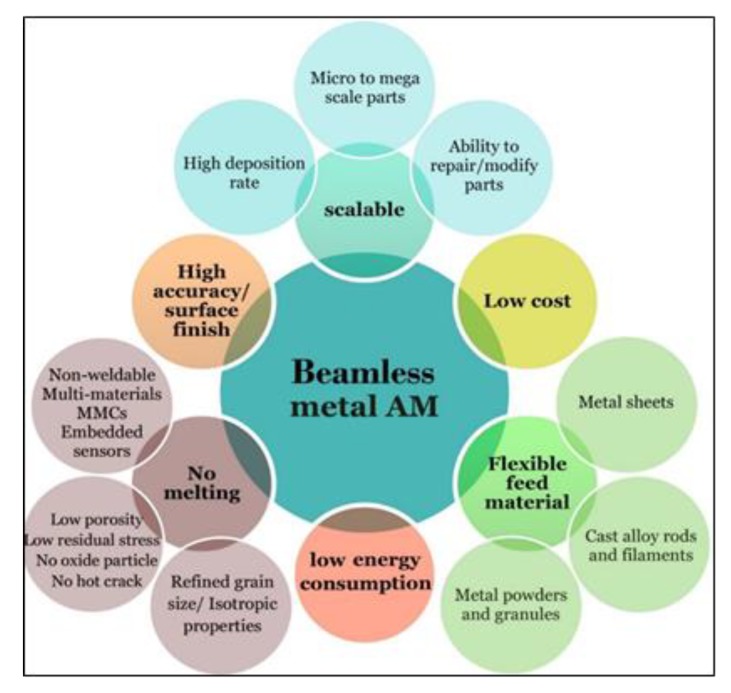
The main possibilities and advantages of the beamless metal-AM over beam-based systems they are low energy, can achieve higher dimensional accuracy, can be used for printing parts from micro to mega scale parts with improved mechanical properties as they do not comprise material melting and rapid solidification.

It is believed that the beamless metal AM can improve the development trajectory of metal AM. It can lower technology and operation costs, overcome the intrinsic safety concerns associated with beam-based systems [4], and possess advantages over beam-based AM technologies as illustrated in Figure 12. The access to cost-effective beamless techniques such as extrusion-based metal AM systems facilitates wider adoption of and a greater business value from this technology. This accessibility allows the small/medium size companies and job shops to enter the metal-AM market whereby they can shorten lead-time, increase their market penetration and share, and expand their product portfolio. In particular, beamless metal AM would hold great potential for less developed countries that possess inadequate production infrastructure. In addition to impact on small public and private sector companies, the large manufacturing companies can also take the advantages of totally new manufacturing possibilities offered by beamless metal AM. For example, AM of high density mega-scale parts is now possible, while it is challenging or impossible for current powder-bed metal-AM systems due to equipment size limitations. Moreover, metal parts with refined grains at higher strength and isotropic mechanical properties are now achievable.

An in-depth understanding of the strengths/weak points and comprehensive knowledge on capabilities and limitations of each beamless metal AM technology is an important step in selecting the appropriate process for a particular application. A comparison of the key beamless metal AM systems has been presented in Table 3.

**Figure 13:**
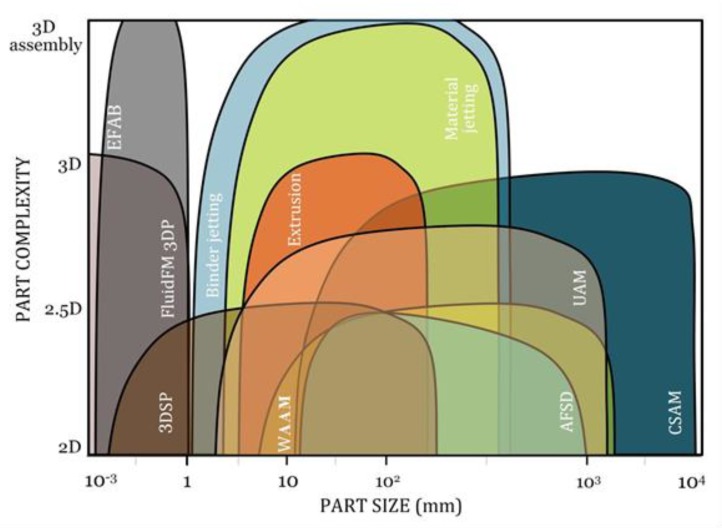
Comparison of the key beamless metal AM systems in terms of part complexity and size. EFAB, 3DSP, FluidFM 3DP are suitable for micro-scale applications. Binder jetting is a scalable process that can be used for micro to medium size parts while material jetting and extrusion methods are normally used for medium size parts. In contrast, CSAM, AFSD, UAM, and WAAW are more suitable for medium to mega-scale parts.

**Figure 14:**
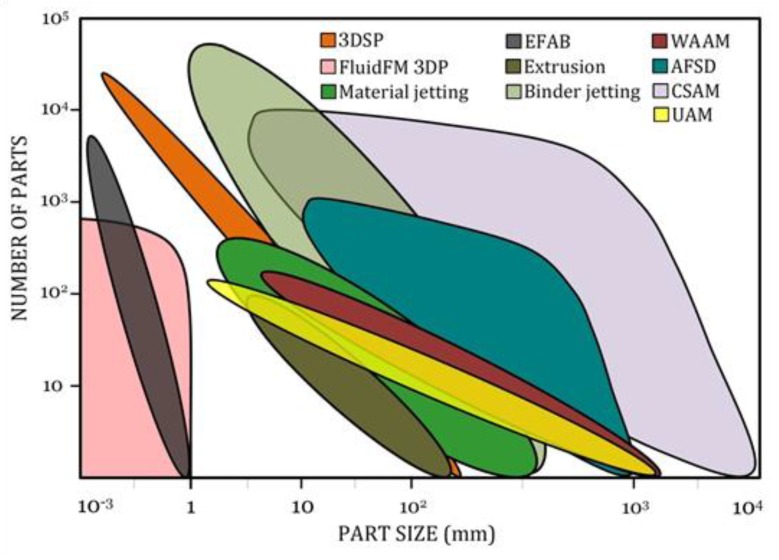
Comparison of the key beamless metal AM systems in terms of part size and productivity. As can be seen, UAM, WAAM, material jetting, extrusion, and FluidFM 3DP are suitable for low-volume production while binder jetting, 3DSP, CSAM, and EFAB have higher deposition rate for high-volume production of metal parts.

**Figure 15:**
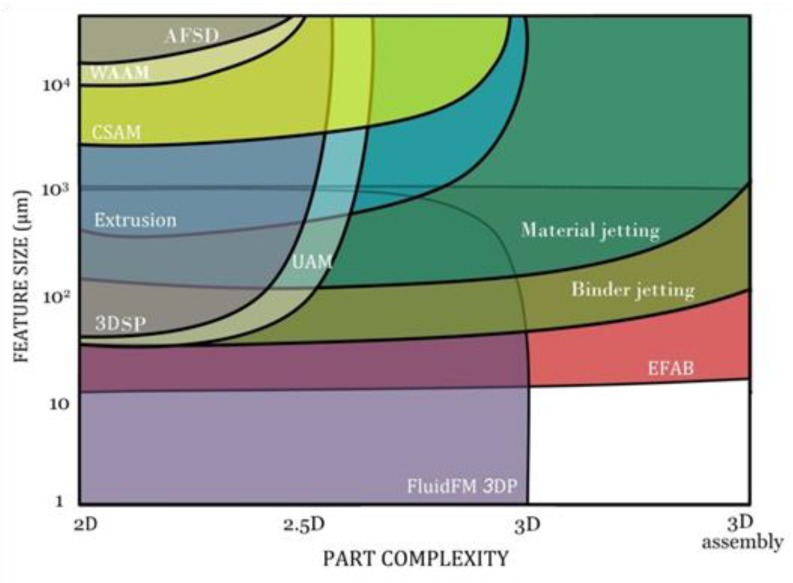
Comparison of the key beamless metal AM systems in terms of part complexity and minimum feature size. Material jetting, binder jetting, and EFAB processes have the capability to print sophisticated 3D parts and assemblies. FluidFM 3DP, extrusion, and CSAM can be used for manufacturing of true 3D parts, while the other processes are normally served for parts with less complexity.

**Figure 16:**
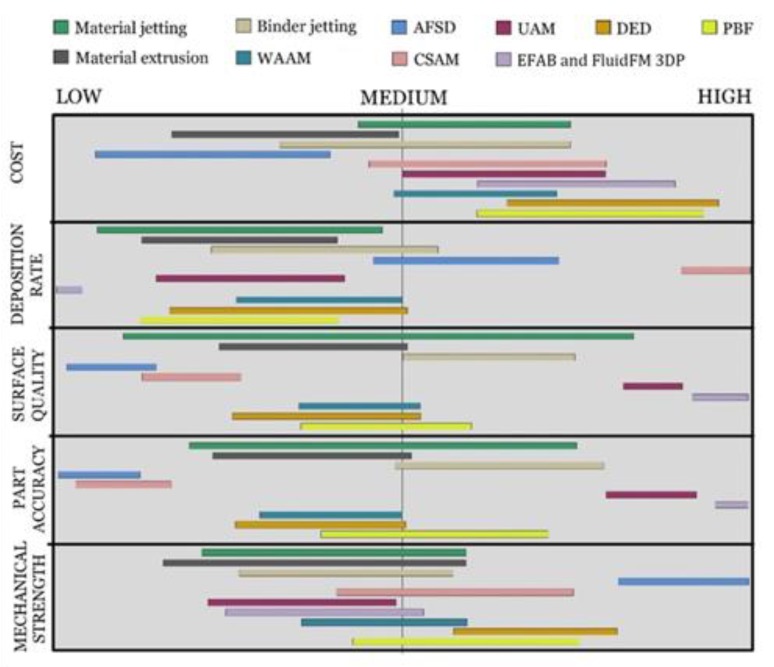
The identification map of the beamless metal AM technologies in comparison with DED and PBF beam-based methods. As can be seen, AFSD offers the highest mechanical strength and the lowest process and operation cost. Extrusion process is also a low-cost process suitable for low-volume production, while CSAM offers the highest deposition rate (up to 38 kg/h). EFAB and FluidFM 3DP processes possess the highest dimensional accuracy and surface quality.

Figure 13, Figure 14 and Figure 15 graphically compare the main beamless metal AM systems in terms of part complexity, minimum feature size, and part size and Figure 16 depicts the identification map of the beamless metal AM technologies.

### 3.2. Application Fields

The application fields of beamless metal AM systems can be classified into three main areas including micro, medium, and mega-scale parts that are discussed in this section.

#### 3.2.1. Micro-Scale Parts

**Figure 17:**
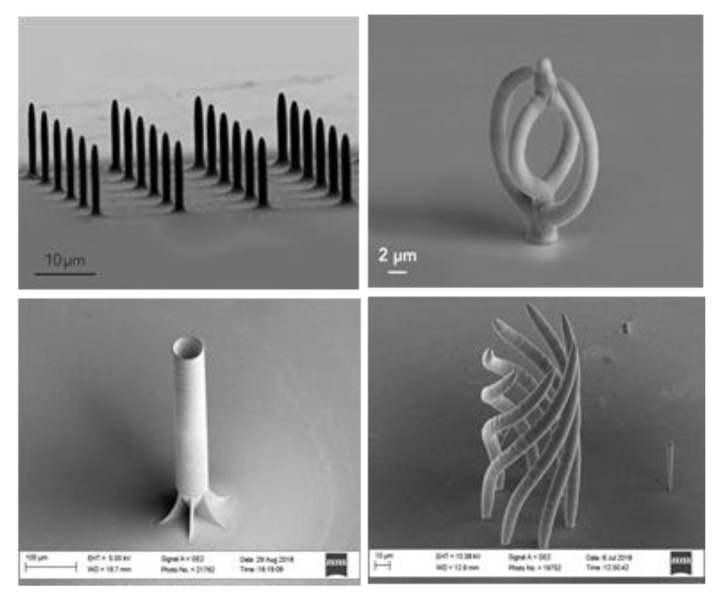
Uniform pillars and complex copper parts printed in single step using FluidFM 3DP without support structure (Courtesy of Cytosurge AG).

The EFAB and FluidFM 3DP are the two current beamless methods that have demonstrated great potential for true 3D micro-manufacturing. The current micro-fabrication techniques normally include multiple production steps which are costly and need strictly safety standards. The FluidFM 3DP is a single step process with potential to overcome these barriers and offer higher efficiency to different micro-scale industries. The size of features printed with FluidFM pinpoint 3DP range from 1 µm to 1000 µm (Figure 17) using a unique 300 nm pipette, allowing flow rates as small as a few femtolitres per second. In particular, the technique allows adding complex parts 3D printed with a high degree accuracy directly onto existing structures or surfaces, on contact pads that are pre-defined on the surface of integrated circuit (IC) boards, on Micro-Electro-Mechanical Systems (MEMS), etc.

The EFAB process has also shown great applicability for true 3D printing of robust sub-millimeter metal parts and mechanical mechanisms for medical, aerospace and semiconductor industries. It provides designers with the new possibilities to commercially produce sophisticated fully assembled medical devices and complex mechanisms with multiple discrete biocompatible components. In particular, as the mechanisms are 3D printed in a finished fully assembled state there is no concern about micro-scale assembly challenges.

**Figure 18:**
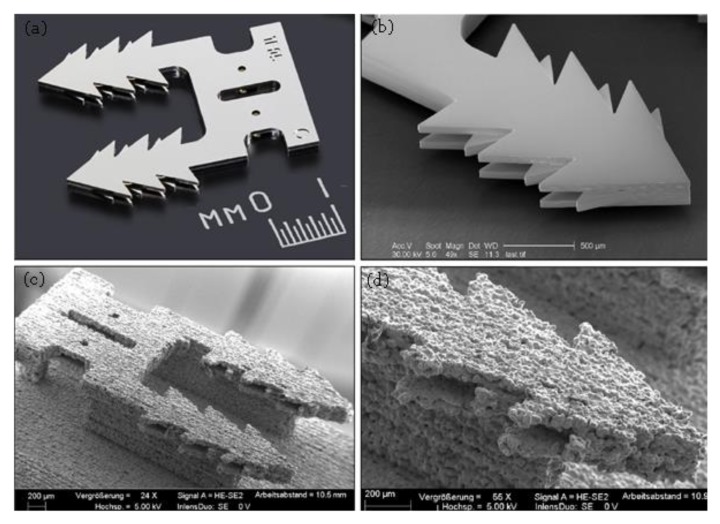
A metal tissue staple 3D printed using (**a**,**b**) EFAB (Courtesy of Microfabrica, USA) and (**c**,**d**) BJ technology (material 316L).

**Figure 19:**
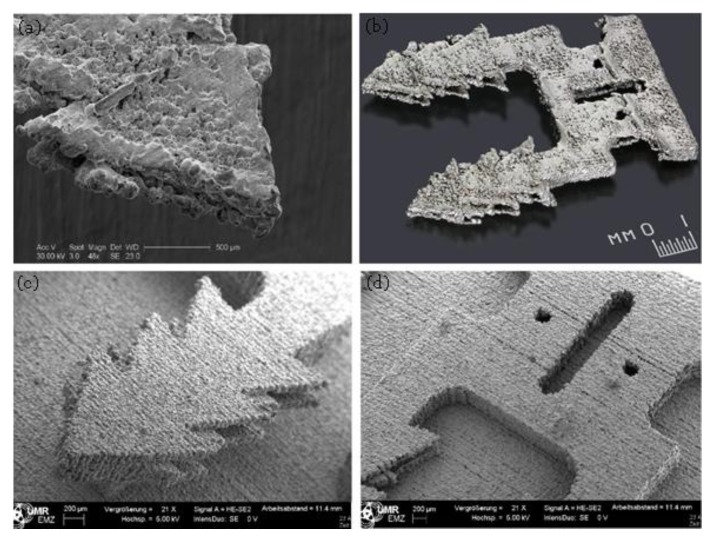
The double enlarged staple (to allow fabrication using beam-based system) printed using (**a**,**b**) SLM process (source: MICA Freeform vs Selective Laser Melting published by Microfabrica, USA) and (**c**,**d**) BJ technology (material 316L).

In addition, proprietary BJ technology is capable of printing very fine parts with micro-features. For better assessment, the authors conducted a benchmarking study. A tissue staple was used as a benchmark sample and printed using Digital Metal^®^ (Höganäs, Sweden) BJ technology at two different sizes to be compared with SLM and EFAB technology. The results of the assessment are presented in Figure 18 and Figure 19.

As can be seen in Figure 18, the printed sample using EFAB technology possesses superior surface finish and accuracy. The lower surface quality and definition of BJ part is apparent, in particular in the teeth region and central locking hole that was designed with undercut features. However, it still proves the capability of BJ to print micro-scale parts that is not possible using SLM or EBM techniques.

**Figure 20:**
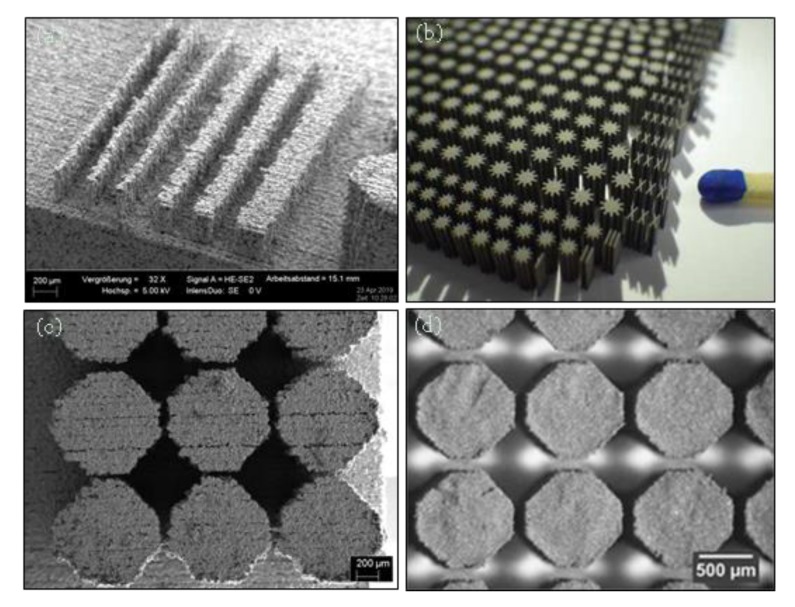
(**a**) Thin walls 316L printed using BJ technology, (**b**) serial production of micro-parts aspect ratio of 1:50 printed using 3DSP [152], (**c**) a benchmarking sample part printed using BJ technology, (**d**) the benchmark sample printed using 3DSP [162].

Figure 19 Compares the double enlarged staple printed using SLM process (Mlab Cusing SLM machine, Concept Laser, Lichtenfels, Germany) and BJ technology (Digital Metal’s 3D printer). Although both SLM and BJ use powder bed technique the BJ part has significantly higher surface quality and part definition. Very thin walls (thickness down to 80 µm) could be printed successfully using BJ process (Figure 20a). So, the BJ process fills the gap between SLM and EFAB technologies in terms of feature size and surface finish. Also, 3DSP process is an emerging technique for serial production of small parts (Figure 20b). The print quality and minimum feature size of 3DSP and BJ falls to relatively similar level. A sample part printed using both techniques is shown in Figure 20c,d. The part produced using 3DSP could achieve a better dimensional tolerance. However, 3DSP cannot compete with BJ technology in terms of part complexity. In 3DSP, a single printing screen is used for a certain number of cycles to create several layers with similar shapes, while many screens must be produced and changed during print for stacking of layers with different shapes that is practically impossible. The process is well suited for mass production of small and simple geometry parts with fine details and walls with high aspect ratios.

#### 3.2.2. Medium Size Parts

Metal BJ is well-suited for truly cost-effective serial production of small to medium size parts for different applications such as medical equipment, portable electronics, luxury watches, etc. that is not currently possible using other beam-based metal-AM systems. Metal BJ possess superior benefits over beam-based powder bed AM systems such as reduced costs with higher manufacturing throughput (as there is no need for support), much less residual stress, increased geometrical complexity and print speed.

Also, BJ process surpasses metal injection molding (MIM) in terms of design flexibility and part complexity, allowing it to penetrate new application fields that are currently not approachable for MIM. Meanwhile, the BJ can complement MIM rather than compete with it. The current MIM producers already have the infrastructure and facilities to process green parts. So, the BJ can be effectively integrated to MIM process for fabricating prototypes and/or small batches before investing in expensive MIM tools for mass production.

Digital Metal^®^’s BJ technology enables detailed fine metal parts to be printed at high volume with remarkably higher precision and surface quality than SLM or EBM processes. The other commercial systems like ExOne (North Huntingdon, PA, USA) and GE Additive (beta H2 binder jetting platform, Cincinnati, OH, USA) have targeted production of medium to large scale parts for automotive and aerospace applications where the BJ technology removes the need for casting and eliminates further expenses such as tooling and molds.

In addition, extrusion-based AM systems can be used for printing of medium size metal parts, in particular, in medical applications such as titanium implants and scaffolds. They can also be served for parts like jigs and fixtures, auto/aero parts, electroforming mandrels, encapsulation molds, dies, electronic joining applications, as well as printing 3D electronic circuitry [82]. Low temperature alloys can be easily processed using direct metal extrusion printing but lack the high mechanical properties of the composite-based FDM processes. With the use of BMGs, the 3D printing process can offer a solution to escape some property-processability tradeoffs.

To date, a large number of both research and commercial works on liquid metal jetting have been demonstrated; however, much of the reports confine to 2D and/or 2.5D printing of low melting point metals for electronics applications [10]. The latest developed liquid metal jetting system (MagnetoJet Technology by Vader Systems, New York, NY, USA) can print medium size parts with arbitrary shapes from low melting point alloys, while lack of support structure remains as the key weak point of the system. In contrast, XJet’s low-temperature nanoparticle inkjet 3D printer can produce complex medium size metal components with high accuracy (dimensional tolerance of 50 to 100 µm depending on part size) and smooth surface finish due to its ultra-thin layer thickness (8–10 µm). Complex 3D geometries and sophisticated assemblies are possible using water-soluble support material. Theoretically, the XJet printed parts have isotropic properties and higher green strength than BJ due to higher level of layer packing.

#### 3.2.3. Mega-Scale Parts

A great focus has been given to the solid-state beamless metal AM technologies for different mega-scale applications in the past few years as reflected by the number of publications [9]. As there is no melting in CSAM, metals are not affected by thermal-related defects and the aluminum or titanium parts do not need to be printed in an inert gas or vacuum sealed chamber, allowing fabrication/repair of much larger components. Also, the CSAM minimizes waste of expensive material by improving the material utilization ratio (buy-to-fly) and machining from near-net-shape parts, not large billets. More important, graded or multiple materials can be effectively printed to tailor functionality of the part.

**Figure 21:**
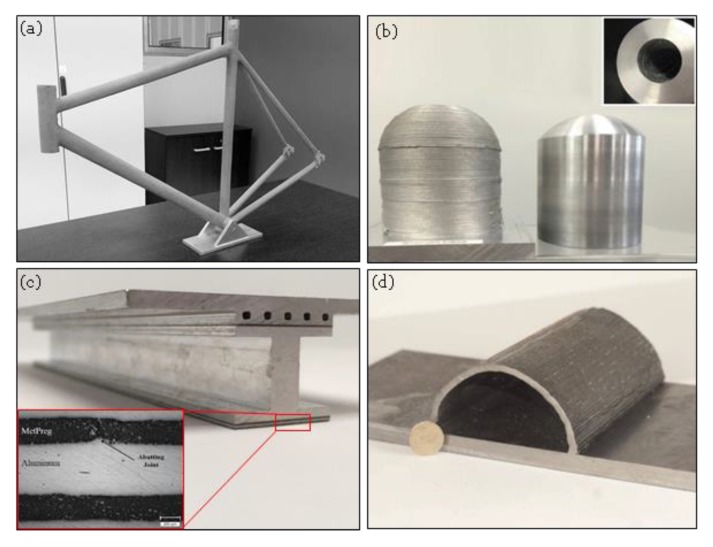
(**a**) A single step 3D printed titanium bicycle frame in 25 min using Titomic’s CSAM system (Courtesy of Titomic, Melbourne, Australia), (**b**) Al6061 hollow dome as fabricated by MELD technology and after finish machining, 115 mm tall, 100 mm diameter part with 25 mm wall thickness, fabrication time: 2 h (Courtesy of Aeroprobe, Christiansburg, VG, USA), (**c**) MMC laminates (MetPreg) composed of continuous alumina fibers and a matrix of pure aluminum are selectively layered for selective reinforcement of the rib structure (Courtesy of Fabrisonic, Columbus, OH, USA), (**d**) 50 mm radius semicircle printed using WAAM [163].

The spot size of the CS process is currently above 4 mm which is significantly larger than other laser-spots in powder-bed AM systems. Therefore, CS-based AM is considered as an ideal choice for cost-effective printing of near-net-shape features onto the available parts. Meanwhile, the CS head can be integrated with an industrial robot arm for super-fast direct printing of very large metal parts with intricate shapes that is not possible using other metal-AM systems (Figure 21a). Titomic (Australia) has commercialized the largest and fastest metal 3D printer with an output size of 9 m × 3 m × 1.5 m for industrial scale production of titanium parts without any shape constrain at ultra–high deposition rate of 38 kg/h.

The other solid-state beamless AM systems, namely, UAM and fiction-based AM systems have been highly regarded by both scientific community and industry. The first commercialized technology of AFSD was developed and patented by Aeroprobe Corporation as “MELD Technology” for printing of a wide range of materials from Al, Mg, titanium alloys, nickel-based superalloys to non-weldable metals at high deposition rates. MMCs can also be deposited through pre-mixing different powders and consolidating them as the feed material. The main application areas of AFSD are repair, rapid manufacture of net-shape large parts (Figure 21b) and production of non-weldable alloys.

UAM has also shown encouraging success in near room temperature fabrication of parts that enables a multitude of thermal-sensitive electronics and sensors to be embedded into the solid metal part, exactly where needed. Also, UAM enables designers to reliably fabricate interwoven channels within a multiple material part printed in a single step. High performance multi-material parts for a wide array of engineering applications, including: coating, heat transfer, strengthening materials, and light weighting can be printed using UAM. To strengthen materials, MMCs can be printed selectively within the part by using continuous fibers at specific high-stress regions (Figure 21c).

In contrast to UAM, WAAM is preferred for the production of even larger parts due to its high deposition rate, high material utilization rate, low production and equipment cost, and high equipment flexibility and scalability. In comparison with subtractive processes, WAAM systems have been shown to reduce fabrication time by 40–60% and post-machining time by 15–20% depending on the component size [145]. Furthermore, the cost of a part produced by the WAAM process is an order of magnitude lower than the cost when produced by laser-based powder processes such as SLM. Applications in the aerospace industry include cryogenic tanks, fuselage, shells and arbitrary profiles (Figure 21d).

## 4. Challenges and Future Perspectives

As a consequence of what was discussed, beamless metal AM is well on its way to becoming a viable and established standard manufacturing tool for cost-effective metal part prototyping and rapid manufacturing. The economic and efficiency advantages ensure that beamless metal AM systems will be subjected to extensive investigations in the future. However, the growth of beamless metal AM has been hampered significantly by technical challenges inherent to each process that are currently limiting the wider adaptation of these promising technologies.

Despite its advantages, metal BJ has been adopted less than beam-based powder bed systems that might be attributed to the inherent porosity present in the parts, even although post-infiltration and/or liquid phase sintering followed by HIP are normally applied to increase density of the parts. In addition, there are some technical challenges preventing wider use of metal BJ. As-printed green parts are relatively fragile which makes them prone to failure, post thermal treatment increases the lead time remarkably, and for larger parts it results in distortion. Future research may be focused on development of novel techniques for enhancing density without sacrificing homogeneity of material, part accuracy, and/or process scalability. Meanwhile it is worth broadening the range of printable materials through the development of new predictive models and optimization of process parameters, and expanding the application field of BJ into composite materials by overcoming powder segregation and co-sintering issues. Moreover, more efforts on high-speed AM (HSAM) using inkjet-based 3D printing systems is expected to be made in the future although the BJ systems possess a higher speed than beam-based powder bed AM systems. This claim has already been fueled by companies such as HP (Palo Alto, CA, USA), and Desktop Metal (Burlington, MA, USA) with prioritizing the print speed in their new metal BJ commercial 3D printers. HP’s Jet Fusion and MultiJet Fusion (MJF) technologies offer high build speed and quality, and at lower cost than competitive 3D printers. These technologies are quite similar to High Speed Sintering (HSS) process [164]. HP’s MJF, uses inkjet printheads to deposit both a fusing agent and a detailing agent onto a bed of powder before sintering them with a set of infrared lamps [165], while HSS does not use a detailing agent. HSS has not been used for true metal 3D printing, while infrared-assisted sintering of inkjet printed metal tracks on substrates have been reported [166]. HP’s Metal Jet printers uses thermal Inkjet printheads to precisely deliver water-based binder to industrial MIM metal powders for reduce costs. It uses multiple printbars (six printheads arranged in two printbars on the print carriage) for high productivity and nozzle redundancy. Desktop Metal’s printer (production model) uses “single pass jetting” technology that employs thousands of piezo nozzles to spray millions of binder droplets per second on the metal powder [167].

The FluidFM 3DP is a newly developed 3D printing method that needs further research and design upgrade to be considered as a mature and reliable technology. The deposition rate needs to be improved for achieving larger build size. Since introduction of the technology the deposition rate of FluidFM 3DP could be increased from 3µm^3^/sec to 375µm^3^/sec for pure metal parts [153]. The increase in printing speed is expected to be considered as a growth strategy. In addition, so far limited metals including copper, silver and gold could be printed, while a great focus should be given on development of other strategic metal systems such as Cd, Cr, In, Zn and Pt in the future.

EFAB process has currently some design constrains such as maximum number of build layers (maximum part height of 1.25 mm) and, more important, parts from very limited metals can be currently manufactured. Smart materials are suggested to be developed in the future for manufacturing of electromagnetism or shape memory-based sensing and actuation systems.

The growth of liquid metal jetting has been hampered by some challenges. The scalloped surface topography of solidified droplets and the staircase effect reduce surface quality. The previous studies on understanding the mechanism of metal droplets impacting and solidification, and optimization of process parameters have been very useful. Further research work should focus on the development of new printing mechanisms, and in-depth understanding of the morphology of surface topography through experiment and modeling would be helpful for adaptation of liquid metal jetting. The low-temperature 3D printing of metallic parts is quite promising. However, it suffers from relatively low print speed (and thus limited build size) due to the use of nanoparticles in thin build layers. In addition, preparation of nanoparticle metal inks and reliable printing of the inks without nozzle clogging and/or particle sedimentation seems to be quite challenging.

CSAM is a vital breakthrough and revolutionary technology and redefines manufacturing components of complex geometry/3D shapes in a single step. Despite the merits highlighted for CSAM, there exist deficiencies that should be addressed through future research activities. The influence of spraying parameters on mechanical properties of hard-to-form materials such as anisotropic behavior is not well understood and entirely agreed upon by researchers. Further experimental studies are required with the aim of developing a standard fabrication strategy for determining optimal part properties. Moreover, most of the research works have focused on the use of CSAM for part restoration and modification, while direct manufacturing of complex parts is an attractive and challenging research topic. In particular, fabrication of parts at higher spatial resolution through development of new spraying systems with smaller spot size for minimizing post-machining processes is a vital step to be further explored.

There are huge application field for friction-based AM in industries where large-scale parts are required such as aviation, defense, automotive, energy, etc. To get the best out of these countless possibilities, development of new practical applications for friction-based AM should be considered as a research focus in the future. The low resolution of material deposition as well as the demand for secondary subtractive machining and/or grinding steps for achieving the final shape are the key barriers to the widespread adaptation of friction-based AM technology for industrial productions. The use of new tool deigns in AFSD with a smaller shoulder diameter might be an effective measure to improve resolution as the amount of deposited plasticized material in each step is decreased. Development of an in-situ quality control system can be another approach to better monitor the FSAM process and enhance resolution and reliability. A closed loop control containing high-speed infrared camera and various sensors is proposed to be used for real-time monitoring of weld formation and in-situ measuring of the peak temperature and cooling rate.

Realization of mentioned potentials for wider applications of UAM is stifled by high material wastage, anisotropic properties and low mechanical strength in build direction, as well as design constrains such as height to width ration. The production cost of UAM parts is also relatively high, and thus the process has been adapted to limited industries such as aerospace or automotive where functionality and/or properties of the engineered multiple material parts can compensate the high costs. The high production cost might be decreased in the feature as metal foils are widely available on the market for prices approaching that of billet. However, the process might not be considered as a route for single material parts fabrication since the high wastage of expensive materials in UAM process remains as the key barrier for cost reduction in the future. The future works should focus on more in-depth investigation of the process parameters affecting ultrasonic welding-induced pores in the interface with the aim of improving mechanical properties. Techniques such as laser-assisted pre-heating of the metal foils might be useful for increasing plastic deformation and thus enhanced bonding.

Many challenges remain with WAAM technology as well. A part from the low resolution of this technology, microstructural anisotropy and porosity remain some of the key challenges that need to be addressed. At the moment, only components with moderate complexity such as flanges or stiffened panel are feasible. Technical challenges like distortion from excessive heat input, relatively poor part accuracy caused by the “stair stepping” effect and poor surface finish keep the technology from being used in more complex and filigree components. The control of residual stresses and distortions especially for the large-scale WAAM process is of major concern. It not only has an effect on the part tolerances but it can also result in premature failure.

The low cost of FDM systems has led to one of the highest adoption rates among AM technologies. However, metal-based FDM systems are not yet developed enough to see widespread use. Further development in print speed through high-aspect-ratio (HAR) nozzles can lead to HSAM of metals [167]. Most approaches with extrusion-based AM with metals use a composite material that needs to be treated after the initial printing process. This can further lead to other tradeoffs such as multi-material implementation or in regard to mechanical properties. The ratio of the contents is also limited to its processability. Frequent buckling failures during the extrusion phase, for example, can cause an interruption of the process. In this context, working with established MIM feedstock holds great promise for the future since a broad range of cost-efficient MIM feedstocks containing various types of metals are already available. Furthermore, it has already been demonstrated that printed parts can achieve mechanical properties that are comparable to values specified for the final MIM parts.

## 5. Conclusions

This paper presented a review on the key beamless metal AM processes, progress and future developments. The sintering-based techniques such as BJ and extrusion-based techniques have demonstrated great potential for cost-effective serial production of small parts and low volume production of medium size parts, respectively. On the other hand, the electrochemical processes such as EFAB and FluidFM 3DP techniques have been able to meet the resolution requirements in micro-manufacturing industry while they provide new possibilities in terms of part complexity. Also, the solid-state beamless techniques provide the opportunity to print mega scale parts with improved mechanical strength. In particular, CSAM process benefits from super-fast metal printing speed, making it well-suited for true industrial production of large parts in the near future.

## Figures and Tables

**Table 1:** Layer bonding mechanisms of different beamless metal AM techniques.

Material Melting	Bulk Sintering	Electrochemical	Thermo/Mechanical
Liquid metal printing, Wire and arc AM (WAAM), Shape Deposition Manufacturing (SDM)	Binder jetting (BJ), Nanoparticles inkjet printing, Material extrusion, 3D screen printing (3DSP), Aerosol jet process, Selective inhibition sintering (SIS)	Electrochemical fabrication (EFAB), FluidFM 3DP	Ultrasonic AM (UAM), Cold spray AM (CSAM), Friction stir AM (FSAM), Additive friction stir deposition (AFSD)

**Table 2:** Comparison of different solid-state beamless metal AM systems [9].

Process	AFSD	FSAM	UAM
ASTM classification	N/A	sheet lamination	sheet lamination
Hybrid process	no	yes	yes
Resolution limiting factor	tool geometry	subtractive process	subtractive process
Temperature	relatively low	relatively high	relatively high
Microstructure	similar to preprocessed	refined, equiaxed in the stir area only	refined, equiaxed

**Table 3:** Comparison of various beamless metal AM processes.

	Advantages		Disadvantages	Metals	Ultimate Tensile Strength (UTS)	Ref
**Extrusion**	Pure Metal	No post-processing required, low-cost hardware, low to no shrinkage during or after additive process	Limited use of metals (low temperature)	Low-melting alloys, bulk metallic glasses (BMG)	Bi_58_Sn_4_2: 51.7 MPa Zr_44_Ti_11_Cu_10_Ni_10_Be_25_: 1200 MPa	[89,94]
	Composite materials	High adoption rate, low cost process, high versatility in materials, isotropic microstructure and mechanical properties, high mechanical properties	Mostly a multi-step process, needs sintering equipment, needs further machining to achieve required tolerances	Stainless steel, Cu, a wide range of metals available as MIM feedstock	316L: 465 MPa (filament) 316L: 524 MPa (MIM feedstock) 17-4 PH: 1.1 GPa (MIM feedstock, hardened) Copper: 6.7 MPa (crushed particles) 17-4 PH: 1.0 GPa (rods)	[90,91] Desktop Metal‘s website
**Material** **jetting**	Liquid metal jetting	Single step process without the need for further post-processing, and thus relatively high production speed	A high temperature melting process, limited to low melting point metals, low surface quality and accuracy, protective build chamber is required, simple geometries without overhang are printable	Low melting point metals such as tin, Cu and Al alloys	7075 Aluminum (373 MPa)	[154]
	Nano-particle ink jetting printing	High accuracy (dimensional tolerance of 50 to 100 µm depending on part size), smooth surface finish due to ultra-thin layer thickness (8–10 µm), low sintering-induced shrinkage and residual stress, complex 3D shapes can be printed using water-soluble support material, isotropic properties and higher green strength than binder jetting due to higher level of layer packing	Relatively low print speed (0.5 to 1 kg/hr) due to fine layer thickness, needs sintering equipment, sintering-induced distortion for large parts, sintering process increases overall production time and cost, difficulties to develop new materials system (as the suitable nanoparticle inks should be engineered), nanoparticle sedimentation and possible nozzle clogging, high technology cost	Ag, stainless steel	No report	XJet’s website
**Binder jetting**	Scalable process (micro to large parts are possible), high accuracy and smooth surface finish (Ra of ~6 µm), high printing speed (0.8–1.5 kg/hr depending on material and layer thickness), lower residual stress than beam-based systems, highly complex 3D shapes and assemblies can be printed without support, relatively low printing cost, suitable for serial production of small parts		High level of porosity (green and sintered density of approx. 50% and 95%, respectively), further heat treatment routes such as infiltration and/or HIP is required, needs sintering equipment, sintering process increases overall production time and cost, sintering-induced distortion for large parts, anisotropic mechanical properties, high technology cost	Cu and Al alloys, stainless steel, titanium, super alloys, iron–manganese alloys, WC-CO hard metals, cobalt-chrome, and magnetic materials	316L (520 MPa, sintering + HIP) SS420(730 MPa, sintering + infiltration with bronze) 17-4PH (900 MPa, sintering + HIP) Ti6-Al-4V (890 MPa, sintering + HIP) Cu (145 MPa, sintered) Cu (176 MPa, sintering + HIP)	[58,71,155]
**CSAM**	A solid-state process without melting, low residual stress, open atmosphere process with very large build area, extremely high deposition rate (up to 38 Kg/hr for titanium), multiple materials and MMcs are possible, very complex geometries can be printed by incorporation of a robotic arm, can be used for both AM and repair applications, microstructure with refined grains		A net-shape process with low accuracy and surface finish, needs further machining to achieve required tolerances, very low spatial resolution (normally 4 mm), needs expensive helium driving gas, low ductility of printed parts, needs further heat treatment to improve ductility	Cu alloys, Al alloys, stainless steel, titanium, and super alloys	Cu (N_2_ driving gas): 220 MPa Cu-Ag-Zr (He driving gas): 500 MPa Ti (He driving gas): 600 MPa Ti-6Al-4V (He driving gas): 765MPa Al7075 (He driving gas): 560 MPa Al6061 (He driving gas): 200 MPa 304L (He driving gas): 420 MPa In718 (He driving gas): 800 MPa	[9,96,104,108,112,113,116,156,157,158]
**UAM**	A low temperature solid-state process, low residual stress, open atmosphere process with large build area, high accuracy and smooth surface finish similar to machining, low energy consumption, very small to large parts are possible, multiple dissimilar material and non-weldable alloys can be printed, different components from optical and SMA fibres to sensitive sensors and electronic devices can be embedded into the parts, mechanical properties of metal foils (feedstock) are retained after printing		A hybrid process with fairly low print speed that needs machining process, high material wastage, anisotropic mechanical properties with considerably lower strength in build direction, high porosity of as-printed parts, design constrains (build height to width ratio), difficult to print complex geometries	A wide range of metals and multi-materials such as Al/Cu, Ni/Stainless steel, Al/Ti, Al/In, Al/Metpreg, Ag/Au, Al/Mo, and Al/Invar	Al-6061 (225 MPa normal to build direction) Al-6061 (46 MPa in build direction) Al-6061 (70 MPa in build direction after HIP)	[128,129,159,160]
**AFSD**	A solid-state process without melting, low residual stress, open atmosphere process with large build area, dissimilar materials, non-weldable alloys, and MMCs can be printed, high deposition rate (up to 9 Kg/h for Al), low operation cost, feedstock flexibility (both metal powder and rod), a single step process (no need for further heat treatment), near wrought microstructure with isotropic and higher mechanical strength than metal bulks due to dynamic recrystallization		A net-shape process with low accuracy and surface finish, needs further machining to achieve required tolerances, low spatial resolution, complex geometries with overhangs can’t be printed	Al alloys, Mg alloys, Cu, steel, Ti alloys, and Ni super alloys, MMCs (Al-SiC, Al-Fe, Al-Mo, Al-CNT, etc.)	Ti-6Al-4V alloy (1.15 GPa) AA5083 (362 MPa) AA6061 (149 MPa) Mg-based WE43 alloy (400 MPa)	[119,141]
**WAAM**	High processing speed (2500 cm^3^/h/ 2–6 kg/h), high material utilization rate, relatively low production and equipment cost, high equipment flexibility and scalability		Low accuracy and resolution (1.5 ± 0.2 mm) microstructural anisotropy and porosity residual stresses and distortions	a wide range of metals	Ti-6Al-4V: 480 MPa (GTAW) 903 MPa (GMAW) Inconel 718: 328 MPa (GMAW) Al:6.3, Cu: 262 MPa	[145,161]
**EFAB**	Very high accuracy and spatial resolution (20 µm), Highly robust microparts and complex mechanisms without the need for assembly can be printed		A multi-step micromanufacturing process with low print speed, limited choice of materials, limited build height (1.25 mm), complete removal of sacrificial material is difficult in some cases	noble palladium, nickel–cobalt alloy, rhodium, Cu	nickel–cobalt alloy: 1.1 GPa	[150]
**FluidFM 3DP**	A single step micro-manufacturing process, very high accuracy and resolution (below 1 µm), complex 3D parts can be printed with pinpoint accuracy directly onto existing structures such as contact pads that are pre-defined on the surface of an integrated circuit (IC) boards, on MEMS, etc.		Limited build height (1 mm), limited choice of materials	Cu, Au, Ag	Not reported	[151]
**3DSP**	Low cost process, relatively high print speed can be achieved, fine to medium size parts can be printed at high resolution (60 µm) and surface finish		A multi-step process, needs sintering equipment, needs screen preparation, simple 2.5D shapes can be printed, further post-heat treatment is required	stainless steel, steel, Cu, a number of different iron-based alloys (17-4-PH)	Not reported	[152,162]
